# Sequential Esophageal Squamous Cell Carcinoma and Small Cell Carcinoma Following Prior Nissen Fundoplication

**DOI:** 10.7759/cureus.108838

**Published:** 2026-05-14

**Authors:** Melika Babadi, Morgan W Stewart, Adil Bhatti, Peter Schultz, Brett Dunbar, Eric Delman

**Affiliations:** 1 College of Osteopathic Medicine, Kansas City University, Kansas City, USA; 2 College of Osteopathic Medicine, Kansas City University, Joplin, USA; 3 General Surgery, Mercy Hospital Pittsburg, Pittsburg, USA

**Keywords:** barrett’s, gerd, immunohistochemistry + ki-67, metachronous dual malignancy, nissen fundoplication

## Abstract

Primary small cell carcinoma of the esophagus (PSCCE) is a rare and aggressive malignancy with clinical features that often resemble more common esophageal cancers, leading to delayed diagnosis and uncertainty in management. We present a unique case of sequential squamous cell carcinoma followed by PSCCE in the distal esophagus after a previous Nissen fundoplication, emphasizing diagnostic and surveillance challenges. A 63-year-old man with a history of gastroesophageal reflux disease (GERD) and prior fundoplication presented with chest pain and progressive dysphagia. Initial esophagogastroduodenoscopy (EGD) revealed a distal esophageal lesion, and biopsy confirmed invasive squamous cell carcinoma arising from Barrett's mucosa, which was treated with endoscopic mucosal resection. One month later, the patient underwent endoscopic ultrasound (EUS) and endoscopic mucosal resection (EMR); the resection specimen showed squamous cell carcinoma in situ without residual invasive carcinoma and had negative margins. Four months after resection, surveillance endoscopy identified a new friable nodular mass in the distal esophagus. Histopathologic evaluation revealed poorly differentiated small cell carcinoma with a high proliferative index, positive staining for synaptophysin and CAM 5.2, and negative staining for chromogranin and CD45. Staging evaluation revealed no evidence of distant metastases.

This report illustrates the rare occurrence of metachronous esophageal cancers with distinct histologic subtypes arising within a short time interval. The rapid development of a second malignancy suggests multifactorial carcinogenesis, including chronic mucosal injury and previous surgical alterations. Given the aggressive nature of PSCCE and the limited evidence available to guide treatment, close surveillance following endoscopic resection of esophageal malignancies is essential, particularly in patients with underlying esophageal pathology. Further studies are needed to better define risk factors and optimal management strategies.

## Introduction

Primary small cell carcinoma of the esophagus (PSCCE) is a rare and aggressive cancer, representing only 0.5-2.8% of all esophageal cancers [1]. Similar to small cell lung cancer (SCLC), PSCCE often exhibits rapid proliferation, early nodal involvement, and metastatic potential [2]. Reported median overall survival ranges from 8 to 28 months [2,3], with isolated five-year survival rates of 6.7-18% despite aggressive treatment [3,4,5]. It most commonly affects older men with a history of tobacco and alcohol exposure, and other risk factors, including chronic gastroesophageal reflux disease (GERD) and Barrett's esophagus (BE), are also recognized [2,4,6]. Early diagnosis is frequently missed because presenting symptoms (i.e., dysphagia, chest pain, weight loss) mimic those of more common esophageal cancers, leading to poor outcomes [2,3,5].

BE is a premalignant condition characterized by the replacement of normal squamous epithelium with intestinal-type columnar metaplasia at the gastroesophageal junction, developing as a consequence of chronic GERD [7]. Nissen fundoplication, the most commonly performed antireflux surgical procedure, effectively controls GERD symptoms; however, evidence suggests that it does not consistently eliminate the risk of neoplastic progression in patients with established BE [8]. Definitive diagnosis relies on endoscopic biopsy with immunohistochemical staining. On histology, neuroendocrine markers such as synaptophysin, CAM 5.2, and CD56 are typically present, along with a markedly elevated Ki-67 proliferative index [2,6,9]. PSCCE is histologically similar to SCLC [10,11].

Treatment options rely heavily on regimens extrapolated from SCLC because there are currently no prospective randomized trials for PSCCE [5]. Limited-stage PSCCE is usually treated with multimodal therapy, including surgery, radiation, and chemotherapy (typically platinum-based), while metastatic disease in the distal esophagus is generally managed with systemic chemotherapy [4,5]. Our case is particularly notable for the metachronous occurrence of squamous cell carcinoma followed by small cell carcinoma in the distal esophagus. Reports of esophageal tumors with distinct histologic subtypes occurring in the same patient are exceedingly rare; one case of synchronous squamous cell carcinoma and small cell carcinoma of the esophagus has been described [12], but to our knowledge, no metachronous sequence has been reported previously. This exceptionally rare sequence emphasizes the need for vigilant surveillance and highlights the diagnostic and therapeutic challenges associated with this aggressive malignancy.

## Case presentation

A 63-year-old man presented to the emergency department in February 2025 with a two-week history of left-sided burning chest pain radiating to the mid-back, accompanied by progressive solid-food dysphagia and partial relief of symptoms with sucralfate. His past medical history was notable for longstanding GERD and a hiatal hernia, for which he had undergone laparoscopic Nissen fundoplication approximately 25 years earlier with sustained symptom control. He also had type 2 diabetes mellitus, diagnosed one year prior, managed with metformin. His social history was notable for extensive tobacco use: he had begun chewing tobacco at age 14, smoked one pack per day from age 30 until quitting five years before presentation, and continued to use approximately one can of smokeless tobacco daily. He reported minimal alcohol intake and had no family history of gastrointestinal malignancy.

**Figure 1:**
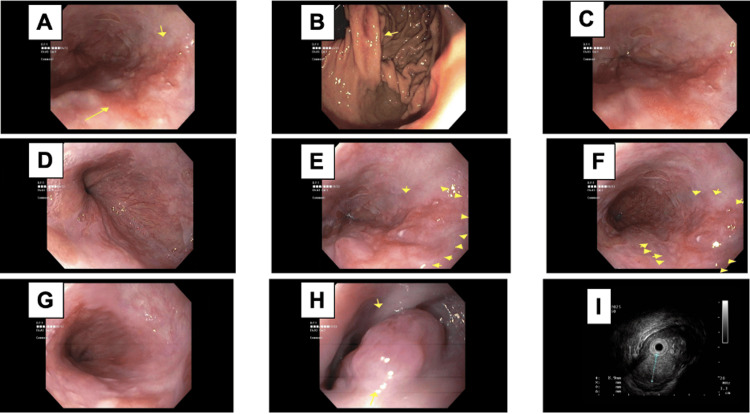
Serial endoscopic evaluations of the distal esophagus across three EGDs In all panels, yellow arrows identify specific mucosal features of interest, and yellow arrowheads delineate the borders of abnormal mucosa. (A-C) EGD1 (February 2025): (A) lower third of the esophagus, with yellow arrows highlighting salmon-colored columnar mucosa consistent with Barrett's esophagus; (B) yellow arrow indicating endoscopic evidence of the prior Nissen fundoplication wrap at the gastroesophageal junction; (C) gastroesophageal junction with subtle mucosal nodularity and irregularity extending from 37-38 cm from the incisors, subsequently shown to harbor invasive squamous cell carcinoma on biopsy. (D-F) EGD2 (March 2025): (D) gastric cardia with Barrett's mucosa before resection; (E–F) abnormal mucosa in the lower third of the esophagus viewed from two different angles, with yellow arrowheads delineating the lesional margins targeted for endoscopic mucosal resection, which was subsequently performed en bloc. The squamous cell carcinoma in situ lesion was located at 37–38 cm from the incisors and was non-circumferential. (G-I) EGD3 (July 2025, surveillance): (G) well-healed post-EMR scar at the Z-line; (H) new friable nodular mass at 34 cm from the incisors, with yellow arrows identifying the lesion; (I) endoscopic ultrasound of the distal esophageal lesion EGDs: esophagogastroduodenoscopies; EMR: endoscopic mucosal resection

**Figure 2:**
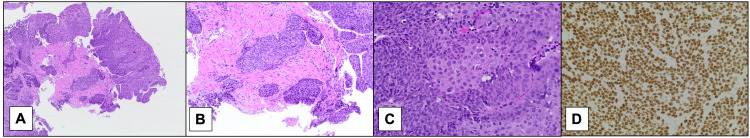
Histopathology of the initial distal esophageal biopsy demonstrating invasive squamous cell carcinoma arising in Barrett's esophagus Histopathology of the initial distal esophageal biopsy, the first esophagogastroduodenoscopy in February 2025, demonstrating invasive squamous cell carcinoma arising in Barrett’s esophagus. (A) Hematoxylin and eosin (H&E) stain, 4× magnification, showing nests of atypical squamous epithelium. (B) H&E, 10×, highlighting stromal invasion by malignant squamous nests. (C) H&E, 20×, demonstrating cellular pleomorphism, hyperchromatic nuclei, and prominent nucleoli. (D) Immunohistochemical stain for p40, 20×, showing diffuse nuclear positivity and confirming squamous cell lineage and ruling out other histologic subtypes

Initial evaluation, including cardiac biomarkers and functional cardiac testing, was unremarkable. Given persistent esophageal symptoms, the patient underwent three serial esophagogastroduodenoscopies (EGDs) over the course of his evaluation (EGD1, EGD2, and EGD3). EGD1 was performed in February 2025 with concurrent dilation, which demonstrated abnormal mucosa in the distal esophagus on a background of salmon-colored columnar-lined epithelium consistent with BE, as well as an intact fundoplication wrap at the gastroesophageal junction (Figures 1A-1C). Targeted biopsies confirmed invasive squamous cell carcinoma arising within Barrett’s mucosa. Histologic examination demonstrated nests and sheets of atypical squamous cells with hyperchromatic nuclei, prominent nucleoli, and evidence of stromal invasion (Figures 2A-2C); immunohistochemistry was diffusely positive for p40, confirming squamous differentiation (Figure 2D).

**Figure 3:**
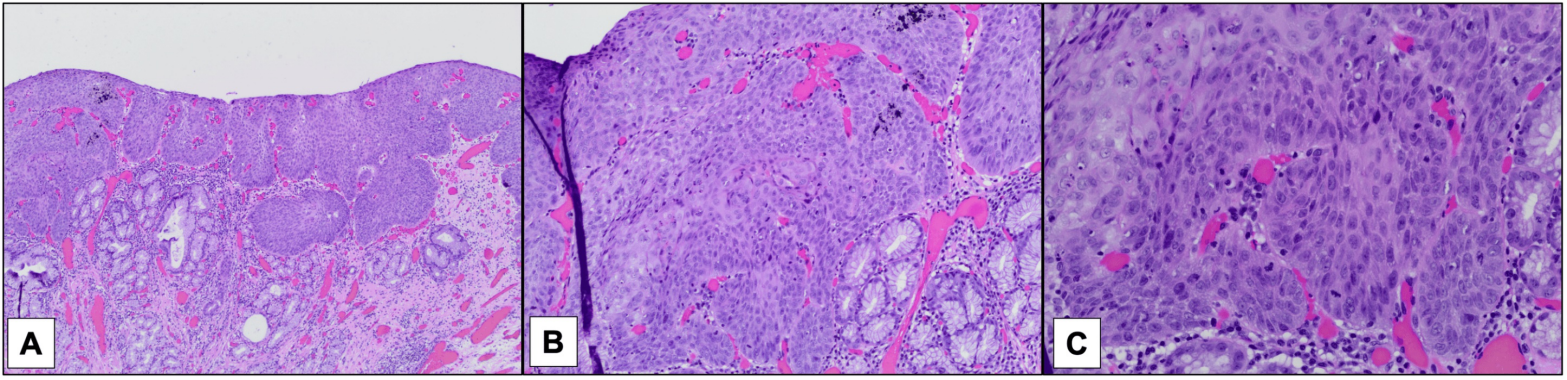
Histopathology of the EMR specimen demonstrating squamous cell carcinoma in situ with negative margins Histopathology of the EMR specimen obtained at the second esophagogastroduodenoscopy in March 2025, demonstrating squamous cell carcinoma in situ without residual invasive carcinoma and negative margins. (A) Hematoxylin-and-eosin (H&E), 4×, showing full-thickness epithelial dysplasia without basement-membrane disruption. (B) H&E, 10×, demonstrating loss of squamous maturation and preserved basement membrane. (C) H&E, 20×, highlighting nuclear pleomorphism and loss of polarity confined to the epithelium EMR: endoscopic mucosal resection

In March 2025, the patient underwent endoscopic ultrasound (EUS) and endoscopic mucosal resection (EMR) of the distal esophageal lesion (EGD2). EUS demonstrated the lesion to be confined to the superficial mucosal layers without invasion beyond the submucosa, and EMR was performed en bloc (Figures 1D-1F). Histopathologic examination of the resection specimen revealed squamous cell carcinoma in situ on a background of BE, with no invasive carcinoma identified and negative lateral and deep margins (Figures 3A-3C). These findings were consistent with complete endoscopic eradication of the invasive squamous component identified on the initial biopsy. Following EMR, the patient recovered uneventfully. He remained symptom-free during the interval period, with no recurrence of dysphagia or chest pain, and continued routine follow-up in accordance with the surveillance schedule.

Surveillance EGD (EGD3) in July 2025, approximately four months after EMR, demonstrated a well-healed post-procedural scar at the Z-line (Figure 1G) but also revealed a new, friable, nodular mass in the distal esophagus approximately 34 cm from the incisors (Figure 1H). Endoscopic ultrasound performed at the same session demonstrated a hypoechoic mass invading beyond the muscularis mucosae (Figure 1I). Biopsies were obtained from both the mass and the gastroesophageal junction.

**Figure 4:**
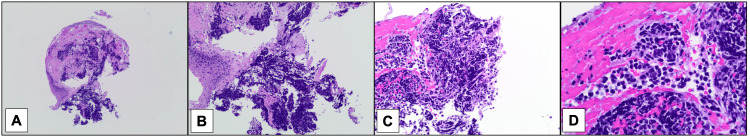
Histopathology of the surveillance biopsy demonstrating poorly differentiated small cell carcinoma of the distal esophagus Histopathology of the distal esophageal mass biopsied at the third (surveillance) esophagogastroduodenoscopy in July 2025, demonstrating poorly differentiated small cell carcinoma. (A) Hematoxylin-and-eosin (H&E) stain, 2×, low-power view showing a fragmented, highly cellular neoplasm. (B) H&E, 4×, demonstrating pronounced crush artifact characteristic of small cell carcinoma. (C) H&E, 20×, showing sheets of small tumor cells with geographic necrosis. (D) H&E, 40×, highlighting nuclear molding, scant cytoplasm, and finely granular chromatin

**Figure 5:**
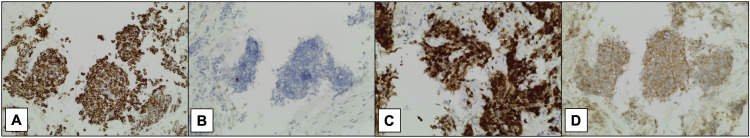
Immunohistochemical profile of the small cell carcinoma confirming neuroendocrine differentiation and high proliferative index Immunohistochemical profile of the small cell carcinoma biopsied at the third esophagogastroduodenoscopy in July 2025. (A) CAM 5.2, 20×, with diffuse cytoplasmic positivity confirming epithelial differentiation. (B) CD45, 20×, showing negative tumor staining and excluding a lymphoproliferative process. (C) Ki-67, 20×, demonstrating a proliferative index exceeding 90%. (D) Synaptophysin, 20×, with diffuse cytoplasmic positivity confirming neuroendocrine differentiation

Histopathologic evaluation of the mass at 34 cm demonstrated a poorly differentiated malignancy composed of small, round-to-oval cells with scant cytoplasm, hyperchromatic nuclei with finely granular (“salt-and-pepper”) chromatin, inconspicuous nucleoli, frequent mitotic figures, extensive geographic necrosis, and prominent nuclear molding (Figures 4A-4D). A pronounced crush artifact characteristic of small cell carcinoma was identified at low power (Figure 4B). Immunohistochemical studies demonstrated diffuse positivity for cytokeratin CAM 5.2 (Figure 5A) and synaptophysin (Figure 5B), confirming both epithelial and neuroendocrine differentiation. The Ki-67 proliferative index was greater than 90% (Figure 5C). Staining for CD45 was negative, excluding a lymphoproliferative process (Figure 5D); chromogranin A was also negative. These findings were diagnostic of poorly differentiated small cell carcinoma. Concurrent biopsy of the gastroesophageal junction (35 cm) demonstrated intestinal metaplasia with acute inflammation, indefinite for dysplasia.

Staging evaluation, including contrast-enhanced CT of the chest, abdomen, and pelvis and PET/CT, demonstrated no evidence of distant metastatic disease or an occult pulmonary primary, supporting a diagnosis of primary small cell carcinoma of the esophagus. The patient was formally staged as T2N1M0 (limited-stage) primary small cell carcinoma of the distal esophagus. After multidisciplinary tumor board review, and in the absence of disease-specific guidelines, management was extrapolated from limited-stage small cell lung cancer protocols.

The patient was initiated on induction chemotherapy with carboplatin and etoposide, followed by concurrent chemoradiation delivered via volumetric modulated arc therapy (VMAT). He tolerated the therapy well, remained clinically stable with resolution of dysphagia, and maintained his performance status throughout the treatment course. He has continued surveillance endoscopy following the completion of the treatment. The most recent surveillance EGD, performed in March 2026, demonstrated benign squamous epithelium with reactive changes and focal chronic inflammation in the distal esophagus, with no evidence of residual or recurrent malignancy.

## Discussion

PSCCE is an extremely rare form of esophageal cancer. Its presentation poses a unique challenge, as the symptoms, such as dysphagia and weight loss, often mimic other esophageal malignancies. Due to its low incidence, no randomized controlled trials exist to define optimal treatment strategies for PSCCE [13]. Chemotherapy regimens are largely extrapolated from SCLC protocols, with current guidelines recommending cisplatin and etoposide [14]. Further research is needed to determine whether this chemotherapy approach is truly optimal for PSCCE or if alternative regimens could provide improved outcomes.

The present case is particularly notable for the sequential development of two distinct esophageal malignancies: squamous cell carcinoma followed by primary small cell carcinoma within a short interval. Reports of metachronous esophageal tumors with different histologic subtypes are exceedingly rare. One possible explanation for this phenomenon is the concept of field cancerization, in which chronic mucosal injury promotes the development of multiple independent malignant transformations within the esophageal epithelium [15]. While metachronous esophageal tumors of the same histologic subtype - particularly multiple primary squamous cell carcinomas - have been reported in the literature with a pooled incidence of approximately 20% following endoscopic or surgical resection, metachronous esophageal tumors with distinct histologic subtypes are far less characterized [16].

In this patient, longstanding GERD and extensive tobacco exposure (both smoking and chewing) may have contributed to a microenvironment conducive to multiple malignant pathways. Another potential explanation is divergent differentiation from a common progenitor cell population within the esophageal mucosa. Regardless of the mechanism, this unusual progression highlights the importance of continued surveillance in patients with risk factors for esophageal malignancy, even after treatment of early-stage disease. 

Given the rarity of PSCCE, no gold standard treatment has been established. The literature is polarized, and consensus on first-line therapy remains lacking. Yan et al. suggested that chemoradiotherapy confers a survival advantage in patients over 60 years of age compared to surgical interventions [17]. In contrast, Cai et al. reported that preoperative chemotherapy combined with surgery improved overall survival in patients with limited-stage PSCCE compared to upfront surgery alone [18]. Similarly, another study found that preoperative radiotherapy and surgery were associated with improved survival [19]. Zhao et al. found that patients receiving concurrent chemoradiotherapy had similar overall survival to those treated with surgery plus chemoradiotherapy, highlighting a potential benefit of concurrent rather than sequential treatment [20]. However, Situ et al. reported no survival benefit from adjuvant chemotherapy in their cohort [14].

As treatment approaches advance, combined modality therapy is thought to improve survival for patients with PSCCE [6]. Future studies should focus on identifying the most effective chemotherapy regimens specifically for PSCCE, evaluating the optimal sequencing of multimodal therapies, and determining which patient populations benefit most from surgery versus chemoradiotherapy. Prospective data collection and multicenter collaboration will be essential to generate evidence-based recommendations.

Although no predisposing risk factors for PSCCE have been identified, some association with smoking, alcohol consumption, and Barrett’s esophagus has been identified [10]. While these factors are not confirmed as definitive risks, they align with our patient’s extensive tobacco use history (one pack per day cigarettes and daily chewing tobacco) and longstanding history of GERD.

## Conclusions

PSCCE is a rare and aggressive cancer with a poor prognosis, further complicated by limited documentation and evidence to guide treatment. This case is unique in that it represents, to our knowledge, the first reported metachronous development of squamous cell carcinoma followed by PSCCE in the distal esophagus, occurring within a four-month interval in a patient with longstanding BE and prior Nissen fundoplication. Notably, adherence to a three-month surveillance protocol following endoscopic resection of the initial squamous cell carcinoma enabled the detection of the PSCCE at a limited stage, underscoring the critical value of close endoscopic follow-up even after successful resection of early esophageal neoplasia. Furthermore, the prior surgical alteration of the gastroesophageal junction and chronic mucosal injury in this patient raise the possibility that Nissen fundoplication, while effective for symptom control, may not eliminate the carcinogenic microenvironment in patients with established BE. Treatment options continue to rely on extrapolated data from SCLC protocols, emphasizing the need for dedicated research into the risk factors, pathogenesis, and optimal management of PSCCE. This report adds to the limited literature on PSCCE and highlights the necessity of vigilant surveillance and continued investigation into this rare and aggressive esophageal malignancy.
